# Hospitals and the Bill

**Published:** 1911-12-02

**Authors:** Wm. Cobbett

**Affiliations:** Chairman. Manchester Royal Infirmary and Dispensary


### The Editor's Letter Box.

### HOSPITALS AND THE BILL.

The secretaries and chairmen of the voluntary hospitals all over the country have shown commendable spirit and energy in supporting the Council of the British Hospitals Association by combining on the lines recommended in The Hospital of October 7, pages 11 and 12. In many cases great hospitals have improved upon the suggestions they received by going further, as in the case of Sir William Cobbett, the Chairman of the Board of Management of the Manchester Royal Infirmary, who has sent a copy of the letter posted to all members of Parliament for the district from which the Manchester Infirmary patients are drawn, and a copy of the resolution arrived at, to the editor of every newspaper circulating in the same area. The following is a copy of Sir William Cobbett's letter to members of Parliament and the resolution:—

Sir,—On behalf of the Board of Management of this Institution I beg to forward the undermentioned resolution of the British Hospitals Association.

The Board are strongly of opinion that the Insurance Bill as it now stands will seriously affect the financial position of voluntary hospitals, and, in consequence, greatly impair both their efficiency and their capacity for good, with the result that insured persons will run grave risks of being deprived of the care and attention in cases of serious illness which can only be obtained by the sick poor of the non-pauper class in voluntary hospitals.

They are strongly of opinion that hospital benefit should be provided by the Bill as well as medical and sanatorium benefits.

They respectfully urge that this most important matter may receive your earnest consideration, and, if possible, your support in Parliament.—I am, Yours faithfully,

Wm. Cobbett, Chairman.

Manchester Royal Infirmary and Dispensary,
November 18, 1911.

Resolution.

That the British Hospitals Association again affirms its unanimous opinion that the National Insurance Bill is incomplete unless it provides for the hospital treatment which insured persons must have if their medical needs are to be covered by the Bill.

[We hope that every voluntary hospital, large and small, which has not yet adopted a similar course will pursue it without a moment's further delay and report the fact to us. We should appreciate the courtesy of any secretary who will send us a postcard stating that his institution is co-operating and has written or is writing to the local M.P.s in support of hospital treatment for the insured.—Ed. The Hospital.]

